# Assessment of needs and gaps in public health cadre in India - a situational analysis

**DOI:** 10.1186/s12913-023-10132-3

**Published:** 2023-10-26

**Authors:** Manish Priyadarshi, Sidharth Sekhar Mishra, Abha Singh, Aanchal Singhal, Moammar Hashmi, Sutapa B. Neogi

**Affiliations:** International Institute of Health Management Research (IIHMR) Delhi, Plot No 3, Sector 18, Sector 18A Dwarka, Phase II, New Delhi, Delhi 110075 India

**Keywords:** Public health cadre, Human resources, Management

## Abstract

**Background:**

A structured and organized public health set up with systematically trained personnel to manage and deliver public health services from grassroot levels to higher administrative levels with separate public health directorate is the need of the hour. The objective of this study was to conduct a situational analysis of public health cadre in select states in India to gain an in-depth understanding of the progress and explore the gaps and challenges in its implementation.

**Methods:**

Four states from the country were selected based on stages of implementation of the cadre. The WHO health systems framework was the basis of assessment. In-depth interviews of 78 stakeholders from public health system across various categories and levels were conducted.

**Results:**

Every state has a dedicated cadre for public health in the form of a separate hierarchical structure and Directorate. There are deficits in human resources skilled enough to manage and implement public health across all levels. Its penetration below districts level is limited. There are limited opportunities available for contractual staffs in terms of remuneration and job progression. The respondents strongly emphasized on having personnel with training in public health, especially at leadership positions. Funding was not reported to be a problem although some challenges in the timeliness of release of funds were reported. Under the existing Health Management Information System, duplication of data exists and there is underutilization of data for policy making.

**Conclusion:**

A dedicated public health cadre is under evolution in India. The main challenge is inadequate workforce skilled in public health management. States are committed to finding solutions to overcome these barriers.

## Background

India has made remarkable progress in public health since independence. There have been significant improvements in health indicators across all the states of the country [1]. However, the health system continues to grapple with several challenges resulting in our inability to meet the targets laid down.

Experiences from developed countries have highlighted the importance of having a structured and organized sector with trained personnel to manage and deliver public health services. Institutionalization of public health delivery systems has resulted in improved population health outcomes [2]. Health systems strengthening is one of the means to progress towards Universal Health Coverage (UHC) [3].

In India, there is a felt need of Public Health Cadre. Public Health Cadre comprises of a systematically structured health care professionals, formally trained in public health, across the health systems starting from grassroot levels to higher administrative levels with separate public health directorate [4–6]. This finds mention in historical documents of Bhore Committee (1946), Mudaliar Committee (1962) and Kartar Singh Committee (1973) [4, 7]. Despite having numerous trained public health functionaries in the country, the system lacks regulatory authority to enforce all interventions and legislation effectively [4]. Creation of a public health cadre with minimal restructuring and disruption of the existing administrative and service delivery structures is a priority of the country. Several meetings and high level discussions have iterated the importance of establishing it by different states [8]. Recent release of Public Health Management Cadre (PHMC) guidelines in the year 2022 reiterates the level of commitment [9, 10]. The guideline spells out the experience and expertise required for various categories of the cadre spanning across the block, district and state levels, for medical, paramedical and non-medical professionals. Medical and health professionals would form a major part, but professionals from diverse backgrounds such as sociology, economics, anthropology, hospital management, communication etc. who have undergone public health management training would also be considered. There is a need to nurture specialized skills like entomology, housekeeping, biomedical waste management, biomedical engineering, communication, management of call centres and ambulance services.

National Institution for Transforming India (NITI) Aayog, a national level think tank urged for introduction of All India and state level public health cadre comprising of public health professionals with multidisciplinary education [1]. Based on the implementation status, the states have been categorized as those having a well-established cadre, those that are evolving in establishing one and those that are in contemplation phase [4]. Though there appears to be a general acceptance, the states are in different stages of introduction of public health cadre [11]. In 2017, the National Health Policy proposed creation of PHMC based on which it was estimated that if implemented from 2020 with roll-out until 2026, then 33,236 posts will be created to serve in the cadre [12]. The PHMC guidelines empower states to develop and implement a roadmap for its implementation [10].

There is little evidence on the situational analysis of public health cadre in India. The renewed commitment to strengthen the cadre calls for a systematic approach to understand the situation [13]. The objective of the study was to conduct a situational analysis of public health cadre in select states in 2020–21, to gain an in-depth understanding of the progress and explore the gaps and challenges in its implementation. This will help understand the facilitators and impediments that are likely to come up while rolling out PHMC in the country.

## Methods

The study was exploratory in nature and utilized the principles of qualitative research. Four states were selected across the country (Gujarat, West Bengal, Odisha, and Maharashtra) based on the level of implementation of the Public Health Cadre and in consultation with the funding partner. Data were collected between April 2021 and January 2022. From each state, ten districts were selected for in-depth interviews of key stakeholders. The districts were selected purposively by the state government based on the geographical location, health profile and feasibility of data collection owing to COVID pandemic. The stakeholders included both officials holding administrative positions in state Governments, districts and non-Governmental sectors, and those involved in implementation of public health services at the district level.

The health systems model proposed by the World Health Organization (WHO) was the framework for the assessment. Information was collected around governance and political will to promote Public Health Cadre, health workforce and financing to support its implementation. Topic guides were developed for data collection and pilot tested, that followed a holistic approach focussing on every element. To maintain the confidentiality, every respondent was given a unique ID. All the interviews were translated into English and then transcribed by the authors who have medical background (without use of any computer/ mobile application). Interviews were audio recorded wherever respondents gave their consent. Based on findings, contextual themes and codes were developed. There was one data coder who provided the codes based on the framework used for the study. Data analysis was done manually using the thematic analysis method. The process of data collection was not iterative.

The interviews were conducted both through online and offline modes, depending on the feasibility due to the pandemic. Those were conducted in English or local language depending on the comfort level of the respondents, by teams of faculty members (with MD or PhD degrees; MP, SSM) and research staffs (with master’s degree in health and hospital management; AS, AS, MH) who were a mix of men (two) and women (two). They had an experience of conducting program relevant research using qualitative techniques. An orientation was provided to all the members on the study protocol.

Each interview (that lasted for 20–40 min) was conducted by a team of two people who were conversant with qualitative research. The interviews were recorded after taking consent from the participants. Privacy was ensured to the extent possible. In case of refusal, the same was recorded through handwritten notes. The names and credentials of the respondent’s record have been kept confidential due to ethical reasons. Data collection was continued till the point of data saturation at every state. The transcripts were not shared with the respondents for their comments and feedback. Repeat interviews were not conducted.

The project was approved by the Institutional Review Board of IIHMR Delhi. The respective states were informed about the study. Permissions were sought from them before start of data collection. We could not obtain permission from one state (Maharashtra) due to constraints resulting from COVID and hence limited ourselves to national level informants with an experience of working in the public health sector of Maharashtra.

Reporting of findings followed Consolidated criteria for Reporting Qualitative research (COREQ) checklist. Data were analysed manually.

### Findings

A total of 78 respondents were interviewed from different states. This included five national experts, eight state officials, 56 district officials, six representatives from development partners, and three retired officials. Two participants refused to participate. The findings emerging from the study have been summarized according to the themes.

#### Political commitment

The first step towards successful implementation of any initiative is ensuring that strategic policy frameworks exist and are combined with effective oversight, coalition-building, regulation, attention to system-design, and accountability.

The national commitment to have a dedicated public health cadre dates back to 1927 – 28, with Tamil Nadu leading and showing the path for rest of India. Currently, all the study states viz. Maharashtra, West Bengal Gujarat, and Odisha have a dedicated cadre for public health in the form of a separate hierarchical structure and Directorate. However, a difference in the nomenclatures exist in the states partly owing to the fact that health is a state subject in India. For instance, it is known as Public Health Cadre in Odisha, Public Health Department in Maharashtra and West Bengal, and public health Division in Gujarat.

At the highest level of hierarchy is the Commissioner Health (an administrative officer). Below that level are Directors representing the Directorates pertaining to Medical Education, Health Services, Administrative services, and Public Health. While Odisha and Gujarat states have a clearly demarcated Public health Directorate with clear cut roles and responsibilities, the same is not true for West Bengal and Maharashtra. Directorate of Health Services oversees the activities earmarked as those of public health cadre in these two states. A respondent mentioned,

“Earlier public health, medical education and medical services were separated but at some point, the health ministry took a decision to merge public health and medicals services and medical education remained as separate because of MCI [Medical Council of India] Regulation.”—P9_PH XVI_RX3_3

Each Director has a team of Deputy Directors to support various activities. Every state has Deputy Directors (below Director Public Health in Odisha and Gujarat and below Director Health Services in West Bengal and Maharashtra) to manage public health activities at the state level and oversee district level activities. The Chief District Medical Officer or Civil Surgeon is the nodal person at the district level. In Gujarat, Additional District Health Officer and in West Bengal deputy Chief Medical Officer Health at district level manages all public health programs who report to the nodal person. In Maharashtra the district is led by two people, one is the civil surgeon responsible for looking after district and subdistrict hospital and other is the district health officer with mandate to have an academic qualification in public health.

To give thrust to the implementation of public cadre in Odisha, restructuring and redesigning has been done at the block, district, and state levels. At the state level, the Special Secretary is in charge of public health supervising all the Directors of Public Health and additional directors. At the district level a special post is created called the Chief District Medical and Public Health officer (CDMO & PHO), who is the nodal person at the district level supported by District Public Health officer (DPHO) and Block Public Health officer (BPHO) at the block level. Such demarcation at the block level was not reported from the states of Maharashtra, West Bengal and Gujarat.

All the respondents substantiated the fact that the state governments are very supportive towards implementation of public health cadre in state.

“Health is a state subject, and we need to focus on Public Health Cadre. We need to create and develop own strategy for implementation of plans.”- P3_PH XVI_RX2_2

All the states reported deficits in having human resources skilled enough to manage and implement public health across all levels. The deficits were more in West Bengal and Odisha. In Gujarat, the respondents reported a high level of attrition in urban areas, perpetuating the problem further. The State health mission provides the varying intensity of training support to personnel responsible for public health. However, there are limited opportunities available for contractual staffs in terms of remuneration and job progression and the biggest fear of lack of job security.

“As public health is big enough so, human resource is little deficient of about 5 – 10%.” A respondent remarked- J3_PH XVIII_RX1_3

The respondents from national and state levels (18) strongly emphasized on having personnel with training in public health, especially at leadership positions. They clearly identified the requirement of having an optimal mix of medical and non medical persons at the helm of affairs. However, a formal training in public health should be a pre-requisite for such positions, the informants expressed.

The national and state level stakeholders lauded the states with a strong public health cadre for effectively managing disasters such as cyclones and pandemics. As expressed by one informant, *“Yes, it a game changer because public health officer monitors and supervises as per the guidelines given by National Health programs. If not supervised, the right outcomes [could] not [be] achieved.”-—T1_PH XVII_RX1_12.*

#### Financing of public health cadre

All the stakeholders echoed that health in India is majorly funded by the central government. An official stated, *“The central government share of the funds is only about 1/3*^*rd*^*, two thirds of the funds for health program comes from the state budget itself, health is a state subject not central.” – P2_PH XVI_RX3_2.*

According to five respondents, if states wish to make a remarkable change in the health system, they must increase their allocations. Approximately 80–85% of the state budget goes into salaries, pensions and maintaining human resources, leaving the rest 10–15% for managing other activities. In fact, revisions of roles and responsibilities in the existing workforce to a certain extent would be enough to undertake the activities expected out of public health cadre. Still fresh recruitments would be required for 10–15% of the vacancies thus created, justifying the need for additional investment, albeit minor.

Three respondents from Gujarat stated that the state budget is optimal but there is a need to create a policy on infrastructure and recruiting sufficient human resources which will help revamp the system. On the other hand, eight respondents from West Bengal and Odisha stated that there are budgetary constraints and state governments are managing with existing resources. Monetary incentives are available for personnel working in difficult terrains. The contractual staffs are underpaid as compared to regular staff and that demotivates them. *“The incentive is for attraction and it’s not performance based, higher the remote location higher are the incentives”, a respondent stated – P3_PHXVI_RX3_4.*

There is no provision for performance-based incentives in Gujarat, Maharashtra, and West Bengal. In Odisha, staffs had received one month salary for their support during the pandemic which is very encouraging. Six respondents also valued the appreciation letter they received from MD as a mark of their contribution to state programs. Expressing anguish over lack of incentives, one respondent mentioned*, “There are so many incentives for our account level and field workers but in case of public health officers there is only public health allowance is there which is bare minimum.” – J3_PH XVIII_RX1_5.*

The state governments are innovating for optimal health finances for their states. For instance, Government of Odisha is taking steps to improve financing through Public Health resource fund (PHRF) that can be easily managed online, as stated by a state official. West Bengal introduced the credit system under NHM for better management of Public Health cadre according to two informants. This ensures every district has fund flow in a smooth manner right from the beginning of the financial year.

#### Role of public health cadre in health care delivery system

The health care delivery system has undergone enormous changes since it was instituted decades ago based on the reports of Bhore committee, Mudaliar committee and Kartar Singh committee, although the basic structure has remained the same. Failure to achieve programmatic targets, despite having a political will and policy guidelines in place, has been attributed to weak managerial skills within the health sector. From being a strictly medical dominated system, it has now expanded to include non-medical professionals into its fold. However, stakeholders believe that the head of the public health cadre should be from medical background, akin to engineering or law departments where the head comes from the same background. *“In law, engineering they are from same background but not in medical sector,”* as mentioned by an informant*- P3_PH XVI_RX2_2.*

Three of the key national and state stakeholders criticized the systems stating that all the decisions are taken at the bureaucratic and political level. They expressed, “*Just putting people in public health cadre will not improve the performance of public health”—P2_PH XVI_RX3_2.*

Every state has a defined Health Directorate and public health cadre with different designations managed by Director Public health or Additional Director public health. The cadre is managed at the state and district levels although the penetration of public health cadre below district level is very limited. Only in Odisha did we observe stakeholders mentioning about creating a sub district level public health cadre. One of the informants from other states highlighted the requirement of technical staff at the block level to enable better decision making.

Another point of concern is resistance from the medical community, for fear of giving up their clinical practice, resistance of allopathic doctors towards traditional system of medicine. One of the respondents opined that for better public health cadre, number of seats for post-graduation in public health in the medical colleges should be increased. Increasing the number of colleges eventually may lead to increase in good quality workforce. Seven respondents stated the need for increasing the intersectoral and interdepartmental coordination of the various departments working along with the public heath cadre creating synergy in the efficient functioning.

#### Public health workforce

An optimally trained workforce is extremely crucial for the successful implementation of Public Health Cadre or Public Health Management Cadre. The cadre comprises of Medical Officers at Primary Health Centres, Community Health Centres, District Hospitals, Public Health Officers and Program/ Health Systems Managers at Block and District levels, Deputy Directors, Joint Directors and Directors at the state level. The Public Health Cadre consists of doctors with specialization in Community Medicine or Public Health. The Public Health Management Cadre is comprised of graduates with post graduation in Public Health (70%), and the remaining are Masters in Business Administration (MBA) in Human resources, Supply chain and procurement, Finance, Operations, Hospital and Health Management (30%). States will have the flexibility to change the proportion as per their needs [9].

All the states studied have a state specific clear cut job description and defined roles and responsibilities for their workforce. In the absence of written guidelines in many states, they generally frame their own rules based on their experience or guidance of their seniors. State specific rules for recruitment are followed in most instances although some respondents reported occasional deviations owing to political reasons.

Shortage of trained professionals were reported from all the states. The reasons reported by almost 50 informants were lack of permanent positions for institutionalizing public health cadre, absence of career progression pathway, disparity in remuneration, especially for those in contractual positions and lack of incentives. The recent pandemic underscored the importance of positions such as epidemiologists, entomologists, and lab technicians in public health cadre.

Career progression is an essential feature to retain skilled professionals which cannot get compensated by incentives alone. Clear-cut career progression, adequate remuneration and increments are defined for permanent staff only. Recognition and awarding deserving positions in the system are equally important to keep the motivation level high. One of the officials also added that there are multiple positions and incremental steps at the bureaucratic level whereas there have been instances where a medical officer is appointed and retires at the same level.

Almost all the respondents opined that there is a need to have non-medical public health professionals in place; having only doctors in public health sector would be a challenge since there is already a dearth of doctors in the country. Moreover, they often lack managerial skills that are important to discharge their duties effectively. Hence, they should mandatorily have public health training that could be at both pre- and in-service levels. Eleven respondents highlighted the challenges in striking a balance between clinical specialists and public health specialists in terms of their promotion and remuneration.

“There should be segregation from the beginning, who will be from the medical side and who will not be from the non-medical side. If you are entering in PH [public health], then you will retire in PH.” A respondent remarked. P5_PH XVI_RX1_3

“Now it has been made mandatory for all Public Health Cadre posts that they must be trained on public health activity either diploma or PG diploma or MPH.”- P7_PH XVI_RX2_5

A lot of emphasis is given to in-service trainings. Trainings ought to be designed and customized in a way that they facilitate career progression. This essentially translates to having recognized courses delivered online or in hybrid mode, that may span one to two years depending on the type of course and the accrediting Institute. Shorter duration trainings ranging from one week to 3 months are useful provided there is a system of cumulative credit transfer, culminating in a diploma.

However, a concern was raised that existing human resources trained in Preventive and Social Medicine in Medical colleges are not being utilized effectively because of their lack of interest and motivation. One suggestion provided by an informant was that *“It was discussed that postgraduates in management can start working at block level, those with any medical degree at the district level and those with medical degree as overall in charge as they have more comprehensive of public health in general.”—P2_PH XVI_RX3_2.*

Mixed responses were obtained when the respondents were asked about the including Accredited Social Health Activists (ASHA), grassroot level functionaries as part of the public health cadre. Most of the informants (56) stated that ASHAs should be the part of the cadre as they are the major driving forces on the field. While others (13) expressed that ASHA should be kept as separate since she is a volunteer worker and receives incentives.

Unlike other states, respondents (10) from Odisha lauded the efforts being put in by the state to capacitate its workforce. The state has collaborated with various institutions to provide one-year courses in public health or diploma or allied courses. As expressed by one respondent:

“The good news is there you might be aware that this year onwards NHM Odisha would be training onwards approx. 200 personnel in each year for the next four years.” -P7_PHXVI_RX1_20

A SWOT analysis of the implementation of Public Health Cadre is provided in Table 1.

**Table 1:** SWOT analysis of the implementation of public health cadre in select states

**Strengths**
• There is a political commitment at the highest level. It finds a mention in documents of National Health Policy, NITI Aayog and National Health Mission
• There is reasonable provision of budget for establishing and maintaining public health cadre
• Every state has a defined structure for implementing public health programs in community and facility
**Weaknesses**
• Lack of a policy document to guide states to create and manage public health cadre
• Plans for capacity building of human resources managing public health cadre, including both pre and in service trainings for medical and non medical professionals needs to reviewed and implemented
• Lack of defined career progression pathways, promotional avenues and opportunities for professionals managing public health cadre
• Absence of a standardized Health Management Information system to yield reliable and accurate data to guide public health programs
• Demarcation of Directorates at the state are defined well but the same at the district levels and below are not too clear
**Opportunities**
• Increasing the accountability of every personnel in public health cadre by defining their roles and responsibilities
• Providing a capsule training to the professionals before joining their respective positions in public health cadre would improve the overall performance
• Under the Department of Human Resources for Health in the Ministry, there should be a nodal person to look after public health cadre
• The Covid pandemic has underscored the importance of having a trained public health cadre at sub-district level
• Strategies to retain experienced and contractual human resources in the system by bringing parity in the salary structure as that of permanent staff or compensating through incentives
**Threats**
• Differences in nomenclatures and lack of uniformity in organizational structures leads to confusion in terms of assessment of implementation of public health cadre
• Lack of synergy between administration (generally headed by bureaucrats) and implementers (technocrats) leads to delay in the implementation of activities and poses a hindrance in inculcating a sense of ownership among stakeholders
• Health being a state subject demands autonomy but external influences and political interference sometimes deters them from taking independent decisions
• High attrition rates due to contractual nature of most of the positions managing public health cadre at district and block levels

## Discussion

An assessment of public health cadre in select states in India reflects their commitment to have a dedicated cadre in the form of a separate hierarchical structure and Directorate. However, there are concerns around having adequate numbers of professionals formally trained in public health across different levels, and their career progression although budgetary provisions did not seem to be major constraints.

A strong health systems management cadre is being recognized as a pillar to strengthen and manage UHC [14]. Presence of well-defined public health structures in Sri Lanka, Bangladesh, Thailand, and Singapore has resulted in improved health indicators in the region. Intersectoral coordination has remained the mainstay for overall development in health care scenario. For over 125 years [15], this commitment to population health in the form of strengthened departments of public health with ring fenced budgets has helped protect its people from exposure to disease, environmental threats, and helped add years to life and life to years. From well-trained health professionals in independent and locally accountable public health teams within local authorities (as in the UK) to the robust engagement of community level workers (as in countries like Thailand), these institutional arrangements for public health delivery have had a significant effect on improving population health outcomes [16–18]. Even in India, despite having a lower than average health expenditure, presence of good indicators consistently in the state of Tamil Nadu has been attributed to the long standing presence of public health cadre [13].

Despite a commitment and having an optimal infrastructure, lack of formally trained public health personnel remains a perpetual problem. Similar concerns are raised from other countries as well. For instance, a need to develop the capacity of health professionals to effectively manage DHS and provide comprehensive multidisciplinary teams services has been reported from Thailand [18]. Shortage of medical staffs to provide comprehensive health care in government health sectors is reported from Sri Lanka [19].

The three-tiered health system has contributed to expansion of public health services in the country. While the onus of delivering health services will be with medical professionals within it, it is essential to attract the growing management professionals to public health to plug these gaps. Keeping in mind the challenges of human resources in India, a roadmap for career and growth opportunities have been laid out [20]. The career progression for each cadre will be distinctive in their own respective streams with inherent flexibility for inter-cadre deputation wherever necessary, if criteria for qualification is met [9]. Limited evidence on the implementation bottlenecks highlights the divided opinion of doctors in this regard [11].

In UK the National Health Services was established in 1948. Professional training in public health included an academic component delivered by either modular ‘day release’ regional consortia programmes or full-time Masters courses. In response to exposure of health systems failures due to communicable disease outbreaks and falling recruitment, the Acheson Report (1988) reconsidered the fundamental role of public health specialists within the NHS [21]. In most parts of UK, most public health specialists currently come from non-medical backgrounds [21]. The 1970’s saw an expansion of academic departments of community medicine within medical schools, and public health teaching began to feature in undergraduate medical curricula.

The key competencies of public health programs have evolved with time and need. Globally the emphasis of Master level programs include epidemiology and research, health services administration and social and behavioral sciences [22]. The competencies usually revolve around three domains of public health practice: health protection, health improvement and service quality [21]. There is also a shift to introduce public health at the bachelor’s level too. Such courses are increasingly becoming popular, primarily in north America and in Europe [23]. This can help create an interest in the discipline and can shape students’ career choices. They can motivate young professionals to pursue higher studies that are more specialized in nature and in key areas such as health policy. However, it is difficult to regulate the increasing number of schools offering accredited graduate programs and that remains a challenge [24].

The HLEG assessed the needs for health sector managerial cadres at block, district and state levels to be over 1·96 lakhs [20]. With the provision of appropriate career paths, these cadres would progress from block to district and then to state and national levels, resulting in better integration and implementation of programs. It recommends availability of 20 regional centres for faculty development and continuing education. Lack of a clear mandate on public health training, shortfall of skilled human resources, lack of educational infrastructure are some of the impediments reported from several states [11].

Our assessment shows that the states are gradually making efforts to have formally trained public health professionals at the district and state levels. The new guidelines will provide impetus to creating a pool of trained resources. However, there are some anticipated challenges faced while attempting to train the cadre. Firstly, there are not enough institutions that can offer health management courses which may be useful for career progression. Secondly, even those institutions that offer such courses, have a limitation in terms of numbers for enrolling candidates for regular on campus courses. Thirdly, only a handful of candidates can be spared by the health system for academic purposes at any given point of time. Fourthly, online courses, though may overcome this issue, may not always be considered equivalent to offline programs; nevertheless, there are strict criteria that must be met with before offering such online degree courses by any accredited teaching Institution.

There is a thrust to capacitate the public health workforce in the country. Implementation of Public Health Cadre is fraught with several challenges. The findings of the study will provide inputs to overcome the potential challenges and pave the way towards smooth execution of the guidelines laid down. Future studies are required to learn good practices from the states that successfully implement them.

### Limitations and strengths

There are certain limitations of this evaluation. The pandemic led to delays in data collection, and some interviews had to be conducted through online mode. Audio recording of the interviews, though mandated by ethical standards, could have led to partial sharing of sensitive facts by stakeholders. Reliability of the information shared by the interviewees could not be validated due to lack of documentation available on the implementation of public health cadre. Despite these challenges, we ensured that there was an adequate representation of stakeholders and states from different categories defined based on the stages of implementation of Public health cadre. There was a good mix of good and poor performing districts, adding to better understanding of the system. Besides, refusal rate was minimum, thus lending to the strengths of the study.

## Conclusion

To conclude, the states have shown a promise to enhance their commitment for strengthening the public health cadre despite several systemic hurdles and challenges. Developing a provision for in-service trainings on management of staff managing public health cadre at district and sub district levels and having a well-defined career progression pathway for those who choose to join public health workforce in the states would lead to greater motivation, and retention of public health professionals. The pandemic has taught us lessons that underscores the need for a strong public health system with skilled and competent workforce to combat any challenges. Any change, big or small requires persistent efforts and perseverance. Post pandemic, with the ever-burgeoning commitment across levels, we are bound to go very far.

## Data Availability

All the data have been retained by the researchers, coded, and anonymized with no direct access provided to the funder. The data that support the findings of this study are available from IIHMR Delhi but restrictions apply to the availability of these data, which were used under license for the current study, and so are not publicly available. Data are however available from the corresponding author (SBN) upon reasonable request and with permission of Thakur Foundation.

## Abbreviations

ASHA: Accredited Social Health Activist
BPHO: Block Public Health Officer
CDMO: Chief District Medical Officer
COREQ: Consolidated criteria for Reporting Qualitative research
DPHO: District Public Health officer
GDP: Gross Domestic Product
HLEG: High Level Expert Group
HMIS: Health Management Information System
IT: Information Technology
NHM: National Health Mission
NITI: National Institution for Transforming India
PHMC: Public Health Management Cadre
PHO: Public Health officer
PHRF: Public Health Resource Fund
SEARO: Southeast Asian Region
UHC: Universal Health Coverage
UK: United Kingdom
WHO: World Health Organization
